# Medical Management of Three Patients with an Acute Type A Aortic Dissection: Case Series and a Review of the Literature

**DOI:** 10.1055/s-0039-1679870

**Published:** 2019-03-08

**Authors:** Khaled Salhab, William Gioia, Andrew P. Rabenstein, George Gubernikoff, Scott Schubach

**Affiliations:** 1 Department of Thoracic and Cardiovascular Surgery, NYU Winthrop Hospital, Mineola, New York; 2 Department of Surgery, Stony Brook University Hospital, Stony Brook, New York; 3 Department of Cardiology, NYU Winthrop Hospital, Mineola, New York

**Keywords:** aortic dissection, Type A aortic dissection, ascending aorta

## Abstract

The model of surgery first and always for Type A aortic dissections has continued to evolve. During the last three decades, various studies have demonstrated that in select patients, surgery should be delayed or avoided. This case series examines three cases in which patients were medically treated. Furthermore, we review the literature and when surgery should be delayed for acute Type A aortic dissections.

## Introduction

Expedient surgical repair is the standard of care in management of acute Type A aortic dissection (TAAD). Traditional teachings cite a mortality rate for Type A dissection approaching 1% per hour, with a 50% mortality rate within 48 hours 1 and a 75% mortality rate at 7 days. 2 3 The most common cause of death is rupture into the pericardium with resultant cardiac tamponade and circulatory collapse. Other complications of this disease include neurologic injury, acute aortic insufficiency, and myocardial ischemia from coronary dissection and visceral malperfusion from distal propagation of the dissection flap. Despite these potentially devastating complications, there are certain groups of patients in which the operative risk may exceed predicted mortality and medical management may be considered. Medical management is centered around supportive care, blood pressure control, and anti-impulse/negative inotropic therapy with β-blockers to decrease shear forces on the aortic intima. Here, we present a small case series of three patients with acute TAADs who were successfully treated with medical management.

## Case Presentation

### Case 1

**Fig. 1:**
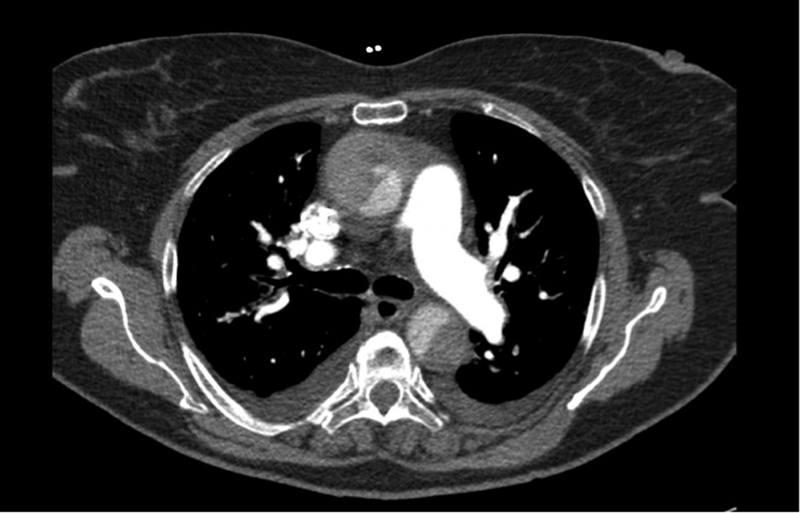
Type A dissection with a definite true and false lumen and an intramural hematoma. The dissection did not extend into the arch vessels; however, it extended down the abdominal aorta. The visceral segment was not compromised, but the left renal artery originated from the false lumen.

A 70-year-old woman with a past medical history significant for hypertension, congestive heart failure, Type 2 diabetes mellitus, hypothyroidism, chronic obstructive pulmonary disease and metastatic colon cancer on chemotherapy presented with acute onset of chest pain. Her medications included multiple antihypertensive drugs—nebivolol, metoprolol, and clonidine. Computed tomographic (CT) angiogram of the chest was performed and demonstrated an acute TAAD with intramural hematoma and a definite true and false lumen ( Fig. 1 ). On presentation to the emergency room, her blood pressure was immediately controlled on nicardipine and labetalol. An arterial line was placed, and the patient was admitted to the cardiothoracic intensive care unit (CT-ICU) for hemodynamic monitoring and blood pressure control. Over the next few days, intravenous (IV) medications were weaned off as the patient was transitioned to oral anti-impulse medications. The patient performed well and was discharged home after 5 days in the hospital. She died 8 months later.

### Case 2

**Fig. 2:**
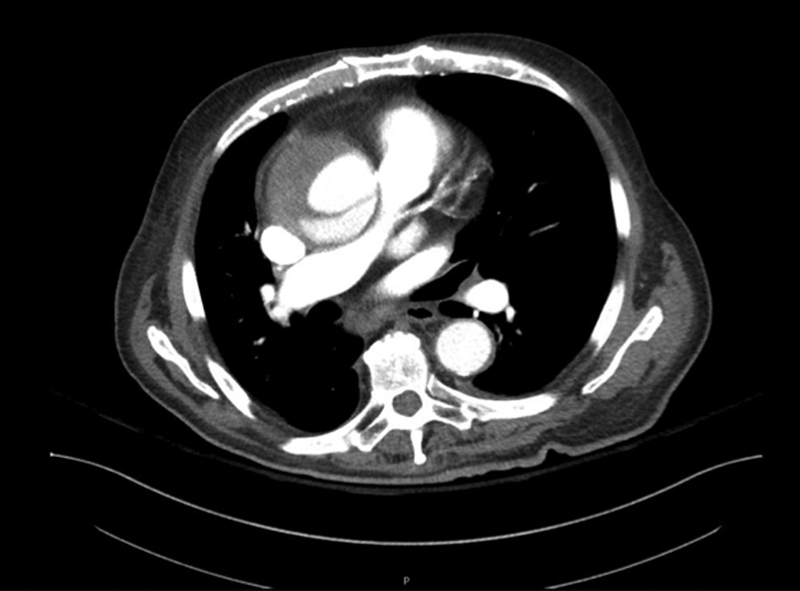
Dissection flap within ascending aortic aneurysm with demonstrated true and false lumens.

An 86-year-old man with a past medical history significant for hypertension, coronary artery disease status post percutaneous coronary intervention (PCI), severe aortic stenosis, status post transcatheter aortic valve replacement (TAVR) 3 years prior, congestive heart failure, atrial fibrillation, and chronic kidney disease presented with worsening dyspnea on exertion, chest pain, orthopnea, cyanosis, and lower extremity swelling. On presentation the patient was noted to be in atrial fibrillation with rapid ventricular response. A CT of the chest was obtained due to the complaint of chest pain. CT at this time showed a 6.7-cm ascending thoracic aortic aneurysm without evidence of dissection. The patient was transferred to our institution for cardiothoracic surgery evaluation of the aortic aneurysm. While hospitalized, he again reported an episode of chest pain. A CT angiogram of the chest was performed and revealed an acute TAAD with a definite true and false lumen ( Fig. 2 ). He was transferred to the CT-ICU and placed on IV anti-impulse medications. Based on his comorbidities, including chronic kidney disease, he was judged to be a poor surgical candidate. Over several days, the patient was transitioned from IV to oral anti-impulse medications and was discharged home. The patient died 1 year later.

### Case 3

**Fig. 3:**
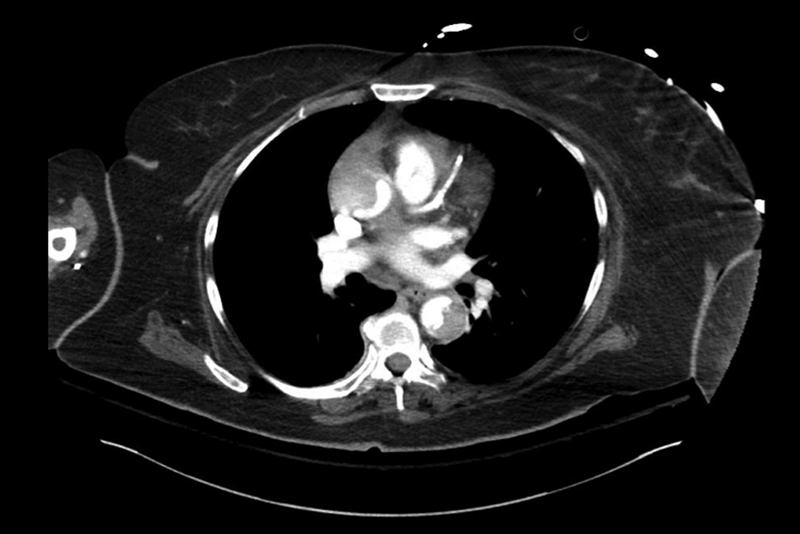
Type A dissection is seen. The dissection extended down the abdominal aorta. The left renal artery was supplied by the false lumen.

A 64-year-old woman with a past medical history of hypertension, coronary artery disease with several episodes of cardiac arrest, bipolar disorder, hepatitis C, hysterectomy, and tracheostomy presented as a transfer from an outside institution for evaluation and management of an acute TAAD that occurred during cardiac catheterization and stenting of the right coronary artery. A CT angiogram was obtained and confirmed an acute TAAD with true and false lumens and a dissection flap extending from the junction of the right- and noncoronary cusps of the aortic valve to the inferior mesenteric artery and left renal artery ( Fig. 3 ). The patient was admitted to the CT-ICU for hemodynamic monitoring and IV anti-impulse control. Because of the patient's comorbidities, the family elected for nonoperative management. Her ICU stay was complicated by respiratory failure requiring intubation and eventual revision of her tracheostomy. During the hospitalization, the patient was diagnosed with sick sinus syndrome requiring a permanent pacemaker. After being transitioned to oral medication, she was discharged to a rehab facility and remains alive.

## Discussion

Over the past 20 years, some studies have aimed to determine whether select groups of patients would have better outcomes with primary medical management of Type A dissection, with or without delayed surgical repair. Feldman and colleagues 4 proposed that patients with completed stroke, advanced age and comorbid conditions, delayed presentation, and prior aortic valve replacement may all warrant initial, if not permanent, medical management of Type A dissection.

A subset of patients who may have an atypical presentation include those who have had previous cardiac surgery. Gillinov et al 5 studied 56 patients with a prior history of cardiac surgery who presented with Type A dissections. Of the 34 patients who presented with acute Type A dissections, only 2 (6%) presented with hemodynamic compromise. Four of six ruptures in this study were contained by adhesions and only identified at surgery, suggesting that prior cardiac surgery may confer a protective effect against life-threatening rupture in some patients.

Though emergent surgery is appropriate for most patients, certain patients may benefit from delayed surgical management. Deeb and colleagues 6 showed that patients with malperfusion have improved outcomes when a strategy of minimally invasive fenestration and medical management is used at presentation. Among those with distal organ malperfusion, 8 (89%) of 9 patients who underwent immediate surgical management died during the same hospitalization whereas 15 (75%) of 20 patients who underwent fenestration combined with medical management and delayed surgical repair survived to discharge. The average delay to surgery was 20 days in this cohort.

The International Registry of Acute Aortic Dissections examined 2,952 patients with acute TAAD. At total 11.1% of patients were treated medically with a 57.1% mortality and 86.4% were treated surgically with 19.7% mortality. 7

If hemodynamics are normal, patients with acute devastating neurologic deficits may also benefit from a period of medical optimization. This strategy combined with intense physical therapy was to the benefit of a 42-year-old man who presented with an acute Type A dissection, right sided hemiplegia and dysarthria. After 5 weeks, the patient was able to regain nearly all of his neurologic function prior to undergoing elective repair. Piccinone et al 8 suggest that this strategy may be used in stable patients, allowing neurologic deficits to plateau prior to major surgery. To the contrary, whereas coma may be seen as a contraindication for operative intervention, a 2006 study by Pocar et al 9 examined five comatose patients who had emergent Type A dissection repair with four out of five patients recovering with no residual defects.

Surgical management has been debated for patients of advanced age. Trimarchi's 2010 study 10 advocated avoiding surgery in octogenarians, as increased age is a strong predictor of in-hospital mortality. Contradictory to these findings, in a study of 24 octogenarians by Tochii et al, 11 the authors concluded that operative management showed better mortality compared with nonoperative management in this age group. These conclusions were echoed in a 2013 study that examined outcomes in 21 octogenarians with Type A dissection. None of these patients experienced in-hospital mortality. 12

Scholl and colleagues 13 examined the outcomes of 34 patients over a 13-year period who underwent delayed operation or no surgery at all. Nineteen out of 34 patients underwent surgical repair more than 48 hours after dissection, and 15 out of 34 never underwent surgery. Survival at 1 to 2 years was better for those who underwent surgery when compared with those who did not (82 vs. 74%), although not statistically significant. This was a surprise to the investigators and led them to suggest that patients who survive the critical period of 48 hours may undergo urgent or semielective surgical repair safely.

In a review of 616 patients with Type A dissection, Centofanti et al 14 found that patients who were treated with medical management alone had a better than expected mortality rate (58%). They suggested that patients with extensive comorbidities and an expected surgical mortality of 58% or greater can be considered for treatment with medical management alone.

The patients in our small case series seem to fit within the current population of patients who are candidates for medical management of Type A dissection. As surgeons, our goal is always to cure with the appropriate surgical intervention, and surgical repair of Type A dissection remains the standard of care. However, patients who survive the initial insult without devastating hemodynamic complications, particularly those with extensive comorbidities, terminal disease, active stroke, and desire to avoid surgery, may be treated medically with delayed repair following optimization or no repair at all. The current literature suggests that a greater than expected proportion of these patients will survive without the need for further intervention.

Emergent surgical management of acute TAADs remains the standard of care in clinical practice. For select patients with advanced comorbidities or a desire to avoid surgery, medical management may be an acceptable initial or permanent option.

## References

[1] Cohn L H Cardiac Surgery in the Adult New York, NY McGraw-Hill 2012

[2] Anagnostopoulos C E Prabhakar M J Kittle C F Aortic dissections and dissecting aneurysms Am J Cardiol 1972 30 03 263 273 4557973 10.1016/0002-9149(72)90070-7

[3] Hirst A E Jr Johns V J Jr Kime S W Jr Dissecting aneurysm of the aorta: a review of 505 cases Medicine (Baltimore) 1958 37 03 217 279 13577293 10.1097/00005792-195809000-00003

[4] Feldman M Shah M Elefteriades J A Medical management of acute type A aortic dissection Ann Thorac Cardiovasc Surg 2009 15 05 286 293 19901881

[5] Gillinov A M Lytle B W Kaplon R J Casselman F P Blackstone E H Cosgrove D M Dissection of the ascending aorta after previous cardiac surgery: differences in presentation and management J Thorac Cardiovasc Surg 1999 117 02 252 260 9918965 10.1016/S0022-5223(99)70420-4

[6] Deeb G M Williams D M Bolling S F Surgical delay for acute type A dissection with malperfusion Ann Thorac Surg 1997 64 06 1669 1675, discussion 1675–1677 9436553 10.1016/s0003-4975(97)01100-4

[7] Pape L A Awais M Woznicki E M Presentation, diagnosis, and outcomes of acute aortic dissection: 17-year trends from the International Registry of Acute Aortic Dissection J Am Coll Cardiol 2015 66 04 350 358 26205591 10.1016/j.jacc.2015.05.029

[8] Piccione W Jr Hamilton I N Najafi H Intentional delayed repair of acute dissection of the ascending aorta complicated by stroke J Thorac Cardiovasc Surg 1995 109 04 807 808 7715232 10.1016/S0022-5223(95)70366-7

[9] Pocar M Passolunghi D Moneta A Mattioli R Donatelli F Coma might not preclude emergency operation in acute aortic dissection Ann Thorac Surg 2006 81 04 1348 1351 16564270 10.1016/j.athoracsur.2005.09.076

[10] Trimarchi S Nienaber C A Rampoldi V Contemporary results of surgery in acute type A aortic dissection: the International Registry of Acute Aortic Dissection experience J Thorac Cardiovasc Surg 2005 129 01 112 122 15632832 10.1016/j.jtcvs.2004.09.005

[11] Tochii M Takami Y Hattori K Early and late outcomes of surgical repair for Stanford A acute aortic dissection in octogenarians Circ J 2016 80 12 2468 2472 27803432 10.1253/circj.CJ-16-0918

[12] Tang G H Malekan R Yu C J Kai M Lansman S L Spielvogel D Surgery for acute type A aortic dissection in octogenarians is justified J Thorac Cardiovasc Surg 2013 145(3, Suppl):S186 S190 23267524 10.1016/j.jtcvs.2012.11.060

[13] Scholl F G Coady M A Davies R Interval or permanent nonoperative management of acute type A aortic dissection Arch Surg 1999 134 04 402 405, discussion 405–406 10199313 10.1001/archsurg.134.4.402

[14] Centofanti P Flocco R Ceresa F Is surgery always mandatory for type A aortic dissection? Ann Thorac Surg 2006 82 05 1658 1663, discussion 1664 17062223 10.1016/j.athoracsur.2006.05.065

